# Nodules de Lisch dans la maladie de Von Recklinghausen

**DOI:** 10.11604/pamj.2014.19.173.5060

**Published:** 2014-10-20

**Authors:** Chama Daoudi, Rajae Daoudi

**Affiliations:** 1 Université Mohammed V Souissi, Service d’‘Ophtalmologie A de l'Hôpital des Spécialités, Centre Hospitalier Universitaire, Rabat, Maroc

**Keywords:** Nodules de Lisch, maladie de Von Recklinghausen, neurofibromatose de type I, Lisch nodules, Von Recklinghausen disease, type I neurofibromatose

## Image en medicine

La maladie de Von Recklinghausen ou neurofibromatose de type I (NF1) fait partie du groupe des phacomatoses. C'est une maladie génétique qui touche autant les femmes que les hommes. Elle résulte d'un désordre précoce de l'embryogenèse. Les lésions provoquées par cette maladie sont très polymorphes, touchant de nombreux organes. Les nodules de Lisch constituent la seule manifestation oculaire de la maladie de Von Recklinghausen. Ils correspondent à des hamartomes mélanocytaires faits de mélanocytes qui contiennent des quantités variables de pigments. Ils sont quasiment pathognomoniques de la neurofibromatose et leur découverte est donc un appoint diagnostique important. Ils sont à distinguer des autres nodules de l'iris: nævus, mélanome, nodules inflammatoires et les anomalies du développement. Les autres atteintes systémiques retrouvées sont de multiples neurofibromes cutanés C'est une patiente âgée de 50 ans, adressée en consultation d'ophtalmologie pour bilan oculaire systématique dans le cadre d'une maladie de Von Recklinghausen. L'interrogatoire retrouve la notion d'antécédents familiaux similaires. La maladie a été révélée par des signes purement cutanés: taches café au lait (A) et neurofibromes multiples (B). L'examen ophtalmologique retrouve une acuité visuelle de 10/10 P2 aux deux yeux. L'examen bio-microscopique du segment antérieur montre de nombreux nodules de Lisch (C) sur les deux iris. Ce sont de petites lésions surélevées par rapport à la surface de l'iris, à bord très net, comme enchâssées dans le stroma, gélatineuses, plus claires que la pigmentation de l'iris, brunes. Elles sont disséminées sur l'ensemble de la surface irienne avec une taille variable. Le fond d'oeil est normal.

**Figure 1:**
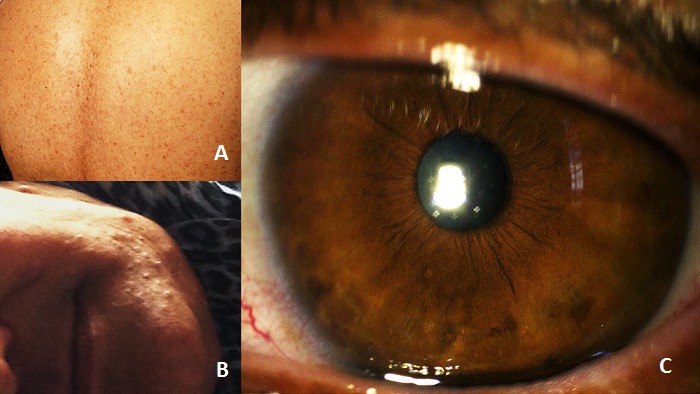
A) taches café au lait; B) neurofibromes cutanés; C) nodules de lisch

